# Predictive Modeling of Vibration Behavior for Ceramic Matrix Composite Thin Plates with Protective Coating in High-Temperature Environments

**DOI:** 10.3390/ma19153296

**Published:** 2026-08-03

**Authors:** Yao Yang, Hui Li, Haijun Wang, Lei Dong, Haile Yan, Haitao Fan, Bingqi Tian

**Affiliations:** 1 School of Mechanical Engineering and Automation, Northeastern University, Shenyang 110819, China; yaoang0716@163.com; 2 Key Laboratory of Vibration and Control of Aero-Propulsion Systems Ministry of Education of China, Northeastern University, Shenyang 110819, China; 3 School of Aeronautics, Shanghai Dianji University, Shanghai 201306, China; navy623@126.com (H.W.); 32121@sdju.edu.cn (H.F.); tianbq@sdju.edu.cn (B.T.); 4 The Third Military Representative Office, Military Representative Bureau of the Naval Equipment Department Stationed in Guangzhou, Guangzhou 510220, China; leidong_1986@126.com; 5 Key Laboratory for Anisotropy and Texture of Materials Ministry of Education of China, Northeastern University, Shenyang 110819, China; yanhaile@mail.neu.edu.cn

**Keywords:** ceramic matrix composite, protective coating, temperature environments, analytical modeling, dynamic characteristics, thermo-vibration resistance

## Abstract

This study proposes a prediction method for ceramic matrix composite thin plates (CMCTPs) with protective coatings under high-temperature conditions, based on the first-order shear deformation theory and the energy principle while considering thermal effects. It can successfully predict the variations in natural frequencies and resonant responses of CMCTPs over the experimentally validated temperature range of 25 °C to 800 °C, using a thermo-vibrational platform with a maximum temperature capability of 1500 °C. In addition, a thorough investigation of multiple key parameter influences on the dynamic characteristics of CMCTPs with and without coating is performed, with special focus on the effect of the coating on enhancing the thermo-vibrational resistance of such structures. The analytical results demonstrate that the protective coating significantly enhances the thermo-vibrational resistance of the structure. Optimization of the coating-to-substrate thickness ratio, elastic modulus ratio, and thermal expansion coefficient ratio is recommended to maximize vibration suppression performance, providing critical guidance for the dynamic design of coated CMCTP components in aerospace.

## 1. Introduction

Ceramic matrix composites (CMCs), renowned for their light weight, high-temperature resistance, and exceptional mechanical strength, have become one of the most popular high-temperature-resistant materials in aero-engine components, thermal protection systems of spacecraft, vehicle brake systems, etc. [1,2,3,4]. In practical applications, CMC components such as blades and panels often operate under severe thermo-mechanical conditions. These conditions include high temperature, high-amplitude vibration, thermal stress, and cyclic mechanical loading, which may cause excessive vibration and fatigue failure [5,6,7,8,9]. To mitigate excessive vibration caused by high-temperature oxidation and thermal shock, protective coatings are needed to extend service lifespan. However, limited research exists on the dynamic behaviors of CMC plates with protective coatings, particularly in high-temperature environments. Thus, a dedicated investigation into their vibration characteristics is essential.

The dynamic behaviors of composite beam, plate and shell structures under different thermal environments have been studied by many scholars [10,11,12,13,14], with current investigations typically combining theoretical modeling and numerical validation. For instance, Shen et al. [15] studied the nonlinear vibration characteristics of nanotube-reinforced composite cylindrical shells under thermal loading conditions (300–500 K), incorporating temperature-dependent material properties in their constitutive modeling. Cunedioglu et al. [16] analyzed the free vibration behaviors of the cantilever glass/epoxy composite beam, spanning from room temperature to 100 °C operational conditions. Marynowski [17] investigated the free vibration behaviors of moving multiscale composite plates under thermal loading (35–200 °C) via the function of reduced frequency depending on the temperature of material properties. Khosravi and colleagues [18] analyzed the effects of uniform temperature elevation (300–1000 K) on the dynamic characteristics of rotating CNT-reinforced composite beam structures. Shi et al. [19] conducted a comprehensive investigation into both the free and transient vibrational responses of functionally graded cylindrical–conical shell structures integrated with annular plates, accounting for coupled thermo-mechanical effects under temperatures of 300 K and 325 K. Based on thermo-elastic theory, Zhong et al. [20] evaluated the stochastic vibration characteristics of laminated combined plate structures, accounting for thermally induced effects with temperature deviations from 0 to 100 K. The aforementioned studies on thermo-vibration analysis have mainly focused on uncoated composite beams, plates, and shells, while the influence of protective coatings has rarely been considered.

Some researchers [21,22,23] have demonstrated the effectiveness of damping coatings in suppressing structural vibrations under various structures and operating conditions. Sun and his team [24] developed an analytical model for hard-coated composite beams incorporating the strain-dependent properties of the coating material to investigate vibration characteristics. Their results demonstrated that the application of a coating significantly reduced the resonance responses of cantilever beam structures. Li and his colleagues [25,26] investigated the vibration suppression performance of two coated composite structures: pyramidal truss sandwich cylindrical shell panels and honeycomb hemispherical shells. Their results demonstrated that protective coatings significantly improved the vibration resistance of both structures. However, these previous studies did not consider the effects of temperature. While his research team has conducted relevant thermo-vibrational analyses of coated cylindrical shell structures, the related study [27] was limited to moderate temperatures below 300 °C and did not address coated CMC thin-walled structures under high-temperature environments.

The literature review shows that the dynamic behavior of the coated CMCTPs under high-temperature conditions has not been sufficiently investigated. To fill this research gap, a novel theoretical model of the CMCTPs with protective coating in a high-temperature environment is proposed in Section 2, which can effectively predict their vibration characteristics. To validate the proposed model, a thermo-vibration coupling system, along with a detailed description of the associated testing procedure, is presented in Section 3. Finally, the effects of several key parameters on the dynamic behaviors of these structures are discussed in Section 4, and a summary of the main research findings is provided in Section 5. The key contributions of this work are as follows: (1) a theoretical model is proposed for predicting the vibration characteristics of CMCTPs with protective coating under high-temperature conditions; (2) the protective coating can improve the high-temperature resistance of CMCTPs; (3) a testing platform capable of 1500 °C is constructed. The present study provides valuable insights into the dynamic behaviors of CMCTPs in high-temperature environments and establishes a theoretical basis for their broader application.

## 2. Theoretical Formulation

### 2.1. Model Illustrations

**Figure 1:**
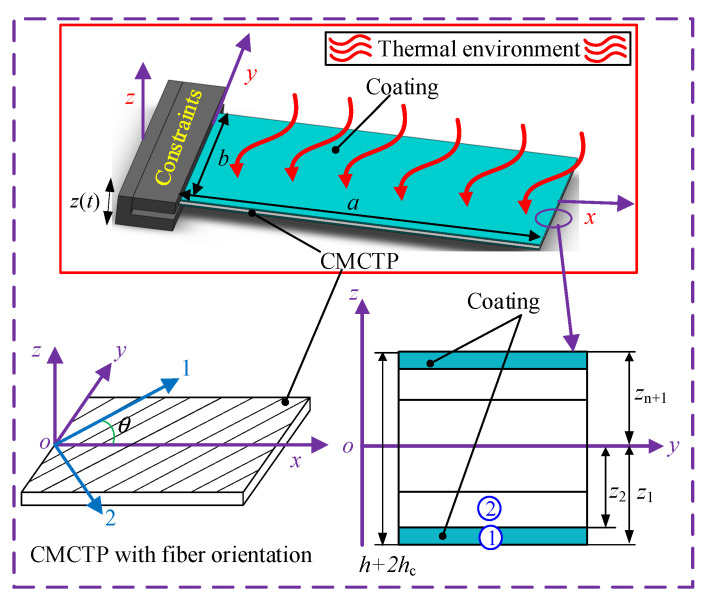
Schematic depiction of CMCTP in a uniform thermal condition.

A dynamic model of CMCTP with the protective coating subjected to a uniform thermal environment (Δ*T*) is developed, as illustrated in Figure 1. The plate’s mid-surface serves as the origin for a Cartesian coordinate *o-xyz*, where *a, b* and *h* represent the length, width, and thickness, respectively. Meanwhile, the plate surface is covered with a protective coating with a thickness of *h*_c_. Assuming the angle between the fiber direction and the *x*-axis direction is *θ*, in this model, CMCTP is composed of *n* layers with the same thickness, and each layer is located at *z*_n+1_ and *z*_n_. Additionally, a local coordinate system (1-2-3) is defined within the fiber layers, where the 1-axis aligns with the fiber direction, the 2-axis is perpendicular to the fibers in the layer plane, and the 3-axis is orthogonal to the 1-2 plane. In addition, the model adopts cantilever constraint in this analysis, with base excitation *z*(*t*) applied at the fixed end. *w*_0_ represents the vibration displacement at point *R*(*x*_1_, *y*_1_). It should be noted that the following assumptions are adopted in the present model to reduce the complexity of the analysis: (1) the coating and structure are rigidly coupled, with no interfacial slip or separation; (2) the temperature field and material are uniform; (3) the nonlinear vibration due to the material nonlinearity of CMC or coating is neglected.

### 2.2. Total Energy Function

The displacement field according to the first-order shear deformation theory [28] can be expressed as:

(1) $$\begin{array}{l} u(x,y,z,t)=u_{0}(x,y,t)+z\varphi_{x}(x,y,t)\\ v(x,y,z,t)=v_{0}(x,y,t)+z\varphi_{y}(x,y,t)\\ w(x,y,z,t)=w_{0}(x,y,t) \end{array}$$

where (*u*_0_, *v*_0_, *w*_0_) denote the components of the mid-surface translational displacement along (*x*, *y*, *z*) directions, respectively. $\varphi_{x}$ and $\varphi_{y}$ represent transverse normal rotation displacements on the *y* and *x* axes, respectively; *t* denotes the time variable.

Consistent with small deformation theory, both displacements and rotations remain sufficiently small to satisfy Hooke’s law. Then, the strains of any point on the CMCTP with protective coatings are given as:

(2) $$\begin{array}{l} \varepsilon_{xx}=\varepsilon_{xx}^{0}+z\kappa_{x},\varepsilon_{yy}=\varepsilon_{yy}^{0}+z\kappa_{y}\\ \gamma_{xy}=\gamma_{xy}^{0}+z\kappa_{xy},\gamma_{xz}=\gamma_{xz}^{0},\gamma_{yz}=\gamma_{yz}^{0} \end{array}$$

Additionally, the mid-surface strain components can be denoted by displacement field variables, which are expressed in the form as:

(3) $$\begin{array}{l} \varepsilon_{xx}^{0}=\frac{\partial u_{0}}{\partial x},\varepsilon_{yy}^{0}=\frac{\partial v_{0}}{\partial y}\\ \kappa_{x}=\frac{\partial \varphi_{x}}{\partial x},\kappa_{y}=\frac{\partial \varphi_{y}}{\partial y},\kappa_{xy}=\frac{\partial \varphi_{y}}{\partial x}+\frac{\partial \varphi_{x}}{\partial y}\\ \gamma_{xz}^{0}=\frac{\partial w_{0}}{\partial x}+\varphi_{x},\gamma_{yz}^{0}=\frac{\partial w_{0}}{\partial y}+\varphi_{y},\gamma_{xy}^{0}=\frac{\partial u_{0}}{\partial y}+\frac{\partial v_{0}}{\partial x} \end{array}$$

where ($\varepsilon_{xx}^{0}$, $\varepsilon_{yy}^{0}$, $\gamma_{xy}^{0}$, $\gamma_{xz}^{0}$, $\gamma_{yz}^{0}$) denote the normal strains and shear strains along corresponding directions, and ($\kappa_{x}$, $\kappa_{y}$, $\kappa_{xy}$) are the curvature changes and twist change at the mid-plane of the structure, respectively.

For the CMCTP with protective coating considered in the present study, the linear thermo-elastic constitutive relations for the CMC substrate and coating under plane- stress conditions are expressed as:

(4a) $$\left.\left\{\begin{array}{c} \sigma_{xx}^{s}\\ \sigma_{yy}^{s}\\ \sigma_{xy}^{s}\\ \tau_{yz}^{s}\\ \tau_{xz}^{s} \end{array}\right.\right\}={\left[\begin{array}{ccccc} {\bar{Q}}_{11}^{s} & {\bar{Q}}_{12}^{s} & {\bar{Q}}_{16}^{s} & 0 & 0\\ {\bar{Q}}_{12}^{s} & {\bar{Q}}_{22}^{s} & {\bar{Q}}_{26}^{s} & 0 & 0\\ {\bar{Q}}_{16}^{s} & {\bar{Q}}_{26}^{s} & {\bar{Q}}_{66}^{s} & 0 & 0\\ 0 & 0 & 0 & {\bar{Q}}_{44}^{s} & {\bar{Q}}_{45}^{s}\\ 0 & 0 & 0 & {\bar{Q}}_{45}^{s} & {\bar{Q}}_{55}^{s} \end{array}\right]}^{\left(k\right)}\left(\left\{\begin{array}{c} \varepsilon_{xx}^{s}\\ \varepsilon_{yy}^{s}\\ \gamma_{xy}^{s}\\ \gamma_{yz}^{s}\\ \gamma_{xz}^{s} \end{array}\right\}-\left\{\begin{array}{c} \alpha_{x}^{s}\\ \alpha_{y}^{s}\\ \alpha_{xy}^{s}\\ 0\\ 0 \end{array}\right\}\Delta T\right)$$

(4b) $$\left.\left\{\begin{array}{c} \sigma_{xx}^{c}\\ \sigma_{yy}^{c}\\ \sigma_{xy}^{c}\\ \tau_{yz}^{c}\\ \tau_{xz}^{c} \end{array}\right.\right\}=\left[\begin{array}{ccccc} Q_{11}^{c} & Q_{12}^{c} & 0 & 0 & 0\\ Q_{12}^{c} & Q_{22}^{c} & 0 & 0 & 0\\ 0 & 0 & Q_{66}^{c} & 0 & 0\\ 0 & 0 & 0 & Q_{44}^{c} & 0\\ 0 & 0 & 0 & 0 & Q_{55}^{c} \end{array}\right]\left(\left\{\begin{array}{c} \varepsilon_{xx}^{c}\\ \varepsilon_{yy}^{c}\\ \gamma_{xy}^{c}\\ \gamma_{yz}^{c}\\ \gamma_{xz}^{c} \end{array}\right\}-\left\{\begin{array}{c} \alpha_{c}\\ \alpha_{c}\\ \alpha_{c}\\ 0\\ 0 \end{array}\right\}\Delta T\right)$$

where $\left(\sigma_{xx}^{s},\sigma_{yy}^{s},\sigma_{xy}^{s},\tau_{yz,}^{s}\tau_{xz}^{s}\right)$ and $\left(\sigma_{xx}^{c},\sigma_{yy}^{c},\sigma_{xy}^{c},\tau_{yz,}^{c}\tau_{xz}^{c}\right)$ represent the stresses of the CMC substrate and coating, respectively. In addition, ${\bar{Q}}_{ij}^{p}(p=s,c;i,j=1,2,4,5,6)$ denote the stiffness coefficients with the superscript denoting the substrate or coating, which is expressed in Appendix A. Δ*T* is the temperature change with regard to a reference state. $\left(\alpha_{x}^{s},\alpha_{y}^{s},\alpha_{xy}^{s}\right)$ and $\alpha_{c}$ denote the thermal expansion coefficients for both substrate and coating materials, in which the coefficients of thermal expansion of CMC in the global coordinate system can be given as $\alpha_{x}^{s}=\alpha_{1}\cos^{2}\theta +\alpha_{2}\sin^{2}\theta$, $\alpha_{y}^{s}=\alpha_{1}\sin^{2}\theta +\alpha_{2}\cos^{2}\theta$, $\alpha_{xy}^{s}=2\left(\alpha_{1}-\alpha_{2}\right)\cos\theta \sin\theta$.

The force and moment resultants of the CMC substrate and the coating are obtained by integrating their respective stress distributions through the plate thickness, yielding

(5a) $$\left[\begin{array}{c} N_{x}^{p}\\ N_{y}^{p}\\ N_{xy}^{p}\\ M_{x}^{p}\\ M_{y}^{p}\\ M_{xy}^{p} \end{array}\right]=\left[\begin{array}{cccccc} A_{11}^{p} & A_{12}^{p} & A_{16}^{p} & B_{11}^{p} & B_{12}^{p} & B_{16}^{p}\\ A_{12}^{p} & A_{22}^{p} & A_{26}^{p} & B_{12}^{p} & B_{22}^{p} & B_{26}^{p}\\ A_{16}^{p} & A_{26}^{p} & A_{66}^{p} & B_{16}^{p} & B_{26}^{p} & B_{66}^{p}\\ B_{11}^{p} & B_{12}^{p} & B_{16}^{p} & D_{11}^{p} & D_{12}^{p} & D_{16}^{p}\\ B_{12}^{p} & B_{22}^{p} & B_{26}^{p} & D_{12}^{p} & D_{22}^{p} & D_{26}^{p}\\ B_{16}^{p} & B_{26}^{p} & B_{66}^{p} & D_{16}^{p} & D_{26}^{p} & D_{66}^{p} \end{array}\right]\left[\begin{array}{c} \varepsilon_{xx}^{0}\\ \varepsilon_{yy}^{0}\\ \gamma_{xy}^{0}\\ \kappa_{x}\\ \kappa_{y}\\ \kappa_{xy} \end{array}\right]-\left[\begin{array}{c} N_{x}^{Tp}\\ N_{y}^{Tp}\\ N_{xy}^{Tp}\\ M_{x}^{Tp}\\ M_{y}^{Tp}\\ M_{xy}^{Tp} \end{array}\right](p=s,c)$$

(5b) $$\left[\begin{array}{c} Q_{x}^{p}\\ Q_{y}^{p} \end{array}\right]=\bar{\kappa }\left[\begin{array}{cc} A_{55}^{p} & A_{45}^{p}\\ A_{45}^{p} & A_{44}^{p} \end{array}\right]\left[\begin{array}{c} \gamma_{xz}^{0}\\ \gamma_{yz}^{0} \end{array}\right](p=s,c)$$

where $\left(N_{x}^{p},N_{y}^{p},N_{xy}^{p}\right)$ and $\left(M_{x}^{p},M_{y}^{p},M_{xy}^{p}\right)$ (p=s,c) denote the internal force and moment components, respectively. $\left(Q_{x}^{p},Q_{y}^{p}\right)$ are the transverse shear force components. $\bar{\kappa }$ denotes the shear correction factor with a value of 5/6. The stiffness coefficients (*A_ij_*, *B_ij_*, *D_ij_*) and thermal internal forces and moments $\left(N_{x}^{Tp},N_{y}^{Tp},N_{xy}^{Tp}\right)$ $\left(M_{x}^{Tp},M_{y}^{Tp},M_{xy}^{Tp}\right)$ can be defined by:

(6a) $$\left(A_{ij}^{p},B_{ij}^{p},D_{ij}^{p})=\int_{h_{p}}{\bar{Q}}_{ij}^{p}(1,z,z^{2})dz(p=s,c;i,j=1,2,4,5,6\right)$$

(6b) $$\left(N_{x}^{Tp},N_{y}^{Tp},N_{xy}^{Tp})=\int_{h_{p}}{\bar{Q}}_{ij}^{p(k)}(\alpha_{x},\alpha_{y},\alpha_{xy})\Delta Tdz(p=s,c\right)$$

(6c) $$\left(M_{x}^{Tp},M_{y}^{Tp},M_{xy}^{Tp})=\int_{h_{p}}{\bar{Q}}_{ij}^{p(k)}(\alpha_{x},\alpha_{y},\alpha_{xy})\Delta Tzdz(p=s,c\right)$$

Based on the definition of kinetic energy, the total kinetic energy *T* of the coated CMCTP is expressed as follows:

(7a) $$T=T_{s}+T_{c}$$

(7b) $$T_{s}=\frac{1}{2}\int_{0}^{a}\int_{0}^{b}\left\{\begin{array}{c} I_{0}^{s}\left({\left(\frac{\partial u_{0}}{\partial t}\right)}^{2}+{\left(\frac{\partial v_{0}}{\partial t}\right)}^{2}+{\left(\frac{\partial w_{0}}{\partial t}\right)}^{2}\right)+I_{2}^{s}\left({\left(\frac{\partial \varphi_{x}}{\partial t}\right)}^{2}+{\left(\frac{\partial \varphi_{y}}{\partial t}\right)}^{2}\right)\\ +2I_{1}^{s}\left(\left(\frac{\partial u_{0}}{\partial t}\right)\left(\frac{\partial \varphi_{x}}{\partial t}\right)+\left(\frac{\partial v_{0}}{\partial t}\right)\left(\frac{\partial \varphi_{y}}{\partial t}\right)\right) \end{array}\right\}dxdy$$

(7c) $$T_{c}=\frac{1}{2}\int_{0}^{a}\int_{0}^{b}\left\{\begin{array}{c} I_{0}^{c}\left({\left(\frac{\partial u_{0}}{\partial t}\right)}^{2}+{\left(\frac{\partial v_{0}}{\partial t}\right)}^{2}+{\left(\frac{\partial w_{0}}{\partial t}\right)}^{2}\right)+I_{2}^{c}\left({\left(\frac{\partial \varphi_{x}}{\partial t}\right)}^{2}+{\left(\frac{\partial \varphi_{y}}{\partial t}\right)}^{2}\right)\\ +2I_{1}^{c}\left(\left(\frac{\partial u_{0}}{\partial t}\right)\left(\frac{\partial \varphi_{x}}{\partial t}\right)+\left(\frac{\partial v_{0}}{\partial t}\right)\left(\frac{\partial \varphi_{y}}{\partial t}\right)\right) \end{array}\right\}dxdy$$

where the kinetic energy terms $T_{s}$, $T_{c}$ and inertia moments ($I_{0}^{s}$,$I_{1}^{s}$,$I_{2}^{s}$), ($I_{0}^{c}$,$I_{1}^{c}$,$I_{2}^{c}$) correspond to the CMCTP substrate and coating components, respectively, which can be written as:

(8a) $$(I_{0}^{s},I_{1}^{s},I_{2}^{s})=\int_{-h/2}^{+h/2}\rho_{s}(1,z,z^{2})dz$$

(8b) $$(I_{0}^{c},I_{1}^{c},I_{2}^{c})=\int_{h/2}^{h/2+h_{c}}\rho_{c}(1,z,z^{2})dz$$

The elastic strain energy $U^{R}$ of the structure at room temperature is expressed as follows:

(9) $$U^{R}=\frac{1}{2}\iint_{A}\left(N_{x}^{p}\varepsilon_{xx}^{0}+N_{y}^{p}\varepsilon_{yy}^{0}+N_{xy}^{p}\gamma_{xy}^{0}+M_{x}^{p}\kappa_{x}+M_{y}^{p}\kappa_{y}+M_{xy}^{p}\kappa_{xy}+Q_{x}^{p}\gamma_{xz}^{0}+Q_{y}^{p}\gamma_{yz}^{0}\right)dxdy,(p=s,c)$$

Accounting for thermal effects, the temperature-dependent potential energy $U^{T}$ can be expressed as:

(10) $$U^{T}=\frac{1}{2}\iint_{A}\left(N_{x}^{Tp}{\left(\frac{\partial w_{0}}{\partial x}\right)}^{2}+N_{y}^{Tp}{\left(\frac{\partial w_{0}}{\partial y}\right)}^{2}+2N_{xy}^{Tp}\left(\frac{\partial w_{0}}{\partial x}\right)\left(\frac{\partial w_{0}}{\partial y}\right)\right)dxdy,(p=s,c)$$

Finally, the work performed by the external excitation forces (*f_u_*, *f_v_*, *f_w_*) is determined as:

(11) $$W_{F}=\underset{V}{∭}\left(f_{u}u+f_{v}v+f_{w}w\right)dV$$

### 2.3. Lagrange Energy Equation

This study employs Jacobi orthogonal polynomials to formulate the mid-surface displacement functions, which can be expressed as:

(12) $$\begin{array}{l} u_{0}=\sum_{m=1}^{M}\sum_{n=1}^{N}A_{mn}P_{m}^{\left(\vartheta ,\zeta \right)}(x)P_{n}^{\left(\vartheta ,\zeta \right)}(y)e^{i\omega t},v_{0}=\sum_{m=1}^{M}\sum_{n=1}^{N}B_{mn}P_{m}^{\left(\vartheta ,\zeta \right)}(x)P_{n}^{\left(\vartheta ,\zeta \right)}(y)e^{i\omega t}\\ w_{0}=\sum_{m=1}^{M}\sum_{n=1}^{N}C_{mn}P_{m}^{\left(\vartheta ,\zeta \right)}(x)P_{n}^{\left(\vartheta ,\zeta \right)}(y)e^{i\omega t}\\ \varphi_{x}=\sum_{m=1}^{M}\sum_{n=1}^{N}D_{mn}P_{m}^{\left(\vartheta ,\zeta \right)}(x)P_{n}^{\left(\vartheta ,\zeta \right)}(y)e^{i\omega t},\varphi_{y}=\sum_{m=1}^{M}\sum_{n=1}^{N}E_{mn}P_{m}^{\left(\vartheta ,\zeta \right)}(x)P_{n}^{\left(\vartheta ,\zeta \right)}(y)e^{i\omega t} \end{array}$$

where $A_{mn}$,$B_{mn}$,$C_{mn}$,$D_{mn}$,$E_{mn}$ are the unknown expansion coefficients of the constructed functions; *ω* denotes the natural frequency; the truncation numbers of the Jacobi orthogonal polynomials are set as *M* = *N =* 8, which have been verified to provide sufficient convergence for the first few modal frequencies and responses. Further, $P_{m}^{\left(\vartheta ,\zeta \right)}(\Phi )$ denotes the mid-surface displacement functions with their detailed expressions as [29]:

(13) $$\begin{array}{l} P_{0}^{\left(\vartheta ,\zeta \right)}(\Phi )=1,P_{1}^{\left(\vartheta ,\zeta \right)}(\Phi )=\frac{\vartheta +\zeta +2}{2}\Phi -\frac{\vartheta -\zeta }{2}\\ P_{m}^{\left(\vartheta ,\zeta \right)}(\Phi )=\frac{(\vartheta +\zeta +2m-1)\left[\vartheta^{2}-\zeta^{2}+\Phi (\vartheta +\zeta +2m)(\vartheta +\zeta +2m-2)\right]}{2m(\vartheta +\zeta +m)(\vartheta +\zeta +2m-2)}\\ \begin{array}{ccc} & & - \end{array}P_{m-1}^{\left(\vartheta ,\zeta \right)}(\Phi )=\frac{\left(\vartheta +m-1)(\zeta +m-1)(\vartheta +\zeta +2m\right)}{m(\vartheta +\zeta +m)(\vartheta +\zeta +2m-2)}P_{m-2}^{\left(\vartheta ,\zeta \right)}(\Phi ) \end{array}$$

where ϑ,ζ > −1, and its values are provided in Ref. [30].

The admissible displacement functions are constructed by combining Jacobi polynomials with boundary functions to satisfy the essential cantilever boundary conditions of the plate. Substituting these displacement functions into the kinetic and potential energy formulations and applying the Lagrange energy formulation yields:

(14) $$\Pi =U^{R}-U^{T}-T-W_{F}$$

Partial differentiation of the Lagrange energy function with respect to each Ritz coefficients following minimum energy principles, which gives:

(15) $$\frac{\partial \Pi }{\partial A_{mn}}=\frac{\partial \Pi }{\partial B_{mn}}=\frac{\partial \Pi }{\partial C_{mn}}=\frac{\partial \Pi }{\partial D_{mn}}=\frac{\partial \Pi }{\partial E_{mn}}=0$$

### 2.4. Prediction and Solution of Vibration Parameters

Based on the Lagrange energy function obtained in Equation (15), the governing equation of motion can be written as:

(16) $$\left(\begin{array}{c} K+i\omega C-\omega^{2}M \end{array}\right)q=F$$

where **K** denotes the stiffness matrix, **M** represents the mass matrix, **q** corresponds to the vector of undetermined coefficients, and **F** signifies the external forcing vector. In addition, the damping matrix **C** is constructed using the Rayleigh damping model [31,32]:

(17) $$\begin{array}{l} C=\alpha M+\beta K\\ \alpha =\frac{2\omega_{1}\omega_{2}(\zeta_{1}\omega_{2}-\zeta_{2}\omega_{1})}{{\omega_{2}}^{2}-{\omega_{1}}^{2}},\beta =2\left(\frac{\omega_{2}\zeta_{2}-\omega_{1}\zeta_{1}}{{\omega_{2}}^{2}-{\omega_{1}}^{2}}\right) \end{array}$$

where *ω*_1_, *ω*_2_ and $\zeta_{1}$, $\zeta_{2}$ correspond to the first two modal frequencies and associated modal damping ratio of the coated CMCTP, respectively. The Rayleigh damping coefficients *α* and *β* are determined from the experimentally identified damping ratios of selected modes.

The natural frequencies of the damping and forcing terms are set to zero (**C** = 0, **F** = 0), reducing Equation (16) to the following form:

(18) $$\left(\begin{array}{c} K-\omega^{2}M \end{array}\right)q=0$$

Assuming that the harmonic base excitation is applied at the clamped end, the base displacement can be expressed as:

(19) $$z_{b}\left(t\right)=Z_{b}e^{i\bar{\omega }t}$$

where $Z_{b}$ and $\bar{\omega }$ denote the base displacement amplitude and excitation frequency, respectively. The corresponding base acceleration is ${\ddot{z}}_{b}\left(t\right)=-{\bar{\omega }}^{2}Z_{b}e^{i\bar{\omega }t}$. In the experiment, the excitation level is prescribed in terms of acceleration amplitude.

For the coated CMCTP subjected to harmonic base excitation, the equation of motion in terms of the Ritz coefficient vector **q**(*t*) can be written as:

(20) $$M\ddot{q}(t)+C\dot{q}(t)+Kq(t)=F_{b}(t)$$

where $F_{b}(t)$ is the equivalent generalized force vector induced by the prescribed base acceleration.

By introducing the modal coordinate transformation:

(21) $$q(t)=\sum_{r=1}^{n}\phi_{r}\eta_{r}(t)$$

where *ϕ_r_* is the *r*-th mass-normalized mode shape vector and *η_r_*(*t*) is the *r*-th modal coordinate. Equation (20) can be transformed into the frequency domain. For harmonic excitation with frequency $\bar{\omega }$, the steady-state modal response amplitude is obtained as:

(22) $$\eta_{r}(\bar{\omega })=\frac{P_{r}(\bar{\omega })}{\omega_{r}^{2}-{\bar{\omega }}^{2}+i2\varsigma_{r}\omega_{r}\bar{\omega }}$$

where $P_{r}(\bar{\omega })=\phi_{r}^{T}F_{b}(\bar{\omega })$ is the generalized modal force induced by the base excitation; $\omega_{r}$ is the *r*-th natural frequency; $\varsigma_{r}$ denotes the *r*-th modal damping ratio.

The steady-state transverse response amplitude at the measured point *R*(*x*_1_, *y*_1_) is obtained by modal superposition:

(23) $$W_{R}(\bar{\omega })=\sum_{r=1}^{n}\Psi_{r}(x_{1},y_{1})\eta_{r}(\bar{\omega })$$

where $\Psi_{r}(x_{1},y_{1})$ is the transverse displacement component of the *r*-th mode shape at the measured point. The resonant response amplitude is then determined from the peak value of $W_{R}(\bar{\omega })$ near the corresponding natural frequency.

## 3. Experimental Validation and Evaluation

### 3.1. Investigational Specimen

**Figure 2:**
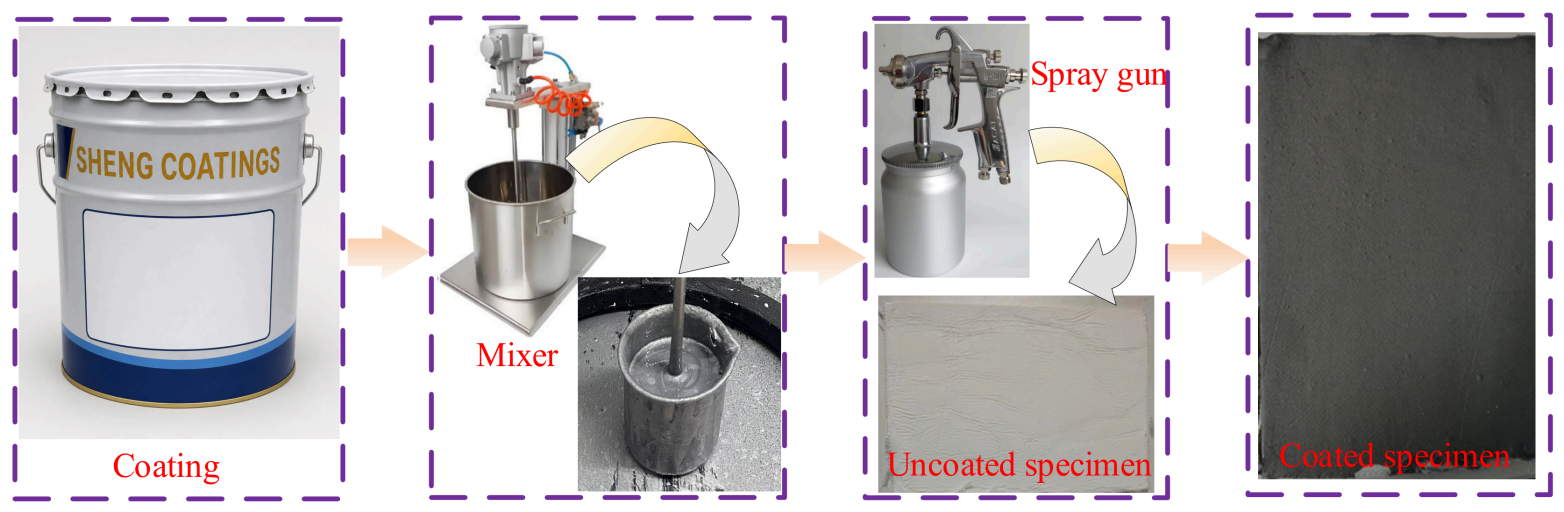
The flowchart of coating deposition on test specimens.

To validate the proposed prediction methodology whose underlying principle is described in Section 2, comparative vibration analyses are conducted experimentally on uncoated and coated CMCTP specimens. Al_2_O_3_-mullite fibrous reinforced composite (Al_2_O_3_-SiO_2_) thin plates are selected as the CMCTP specimens for this investigation. Four specimens are prepared from manufacturer-supplied laminates (Henan Dexian New Material Technology Corporation, Zhengzhou, China) through cutting and surface polishing. All specimens comprise five orthogonally stacked layers in a [0°/90°/0°/90°/0°] symmetric configuration with uniform thicknesses. The custom-developed high-temperature-resistant damping coating is uniformly applied to the specimen surfaces using an air spray gun under precisely controlled deposition conditions (spray pressure: 0.3–0.5 MPa, stand-off distance: 15–20 cm, nozzle diameter 0.5 mm), resulting in a nominal coating thickness of 0.3 mm, as illustrated in the process flowchart in Figure 2. The protective coating used in this work is a ceramic-based high-temperature-resistant damping coating, composed primarily of alumina–silica ceramic constituents and an inorganic high-temperature binder. In the present model, the coating is assumed to have a uniform thickness and to be perfectly bonded to the CMCTP substrate. However, these coating-related properties are not independently characterized through dedicated experiments, such as measurements of thickness uniformity, adhesion tests, microscopic examination of the interface, or post-exposure microstructural characterization. Therefore, the potential effects of local thickness variations, imperfect adhesion, interfacial damage, or coating degradation after prolonged thermal exposure are not considered in the current formulation. The key material properties of the CMCTP substrate are provided by the manufacturer, whereas the thermo-mechanical parameters of the self-designed protective coating are accurately acquired through commissioned evaluations by a professional testing institution. All relevant material properties are listed in Table 1.

### 3.2. Experimental System and Approach

**Figure 3:**
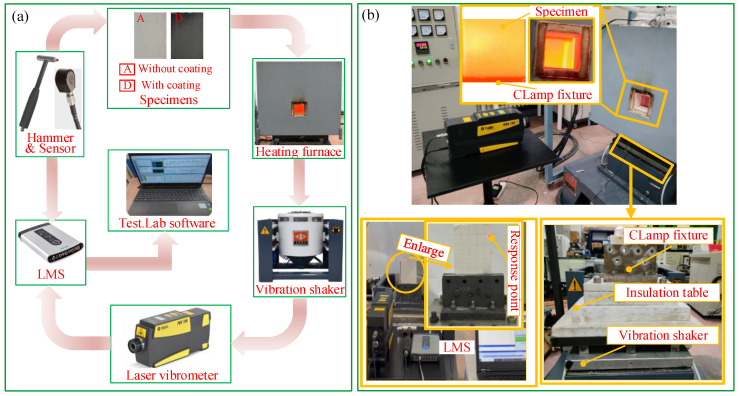
The experimental system: (**a**) testing procedure flowchart, (**b**) vibration test for the specimens.

A specialized high-temperature vibration testing apparatus is developed to evaluate the dynamic response of the specimens, with the complete experimental setup illustrated in Figure 3. The experimental system integrates a controlled ultra-high-temperature furnace equipped with nine molybdenum heating rods and PID thermal regulation, coupled with NiSi-Cr thermocouples for real-time temperature monitoring. Vibrational excitation is delivered via an electromagnetic shaker (Dongling ES-6-230, Suzhou Dongling Vibration Test Instrument Co., Ltd., Suzhou, China), while a laser Doppler vibrometer (model PDV-100, Polytec GmbH, Waldbronn, Germany) enables non-contact velocity measurements. Data acquisition is performed using a Siemens LMS SCADAS system (Siemens Industry Software N.V., Leuven, Belgium) synchronized with a mobile workstation, and the velocity data acquired via PDV-100 are converted to displacement employing Simpson’s first-order numerical integration rule, implemented within Siemens Testlab 2506 software (Siemens Digital Industries Software, Leuven, Belgium). Additional components include a modal hammer (model 086C01, PCB Piezotronics, Inc., Depew, NY, USA) for impact testing, lightweight accelerometers (Type 4517, Brüel & Kjær Sound & Vibration Measurement A/S, Nærum, Denmark), high-temperature-resistant clamping fixture, and modular thermal insulation to ensure experimental stability. Notably, such a system is capable of generating extreme thermal conditions, reaching 1500 °C.

To establish cantilever boundary conditions, specimens are rigidly clamped across a 20 mm width of their long edge using thermally insulated fixtures. The resulting free vibration area maintains dimensions of 150 × 125 × 2 mm. First, impact tests are carried out for the uncoated and coated specimens using a modal hammer to acquire frequency response functions (FRFs) over a frequency range of 0–1024 Hz. Modal parameters are subsequently extracted through spectral analysis, with natural frequencies identified via peak-picking from the FRF magnitude spectra and mode shapes derived using the PolyMax algorithm (Siemens Testlab 2506 Software). Then, the above specimens are subjected to harmonic excitation at the first three natural frequencies (identified via modal analysis) using a vibration control system. Each resonant test condition maintains a 2.0 g excitation amplitude for consistent dynamic response characterization. Subsequently, the modal vibration responses for the first three modes are measured using PDV-100 laser vibrometry under resonant excitation conditions. Notably, vibration responses are acquired at the reference position *R* = (120, 100) for all test specimens. Finally, thermo-vibrational characterization of the other specimens at 400 °C and 800 °C preserves the original test parameters to enable direct performance comparison.

### 3.3. Comparison of Experimental and Theoretical Results

**Figure 4:**
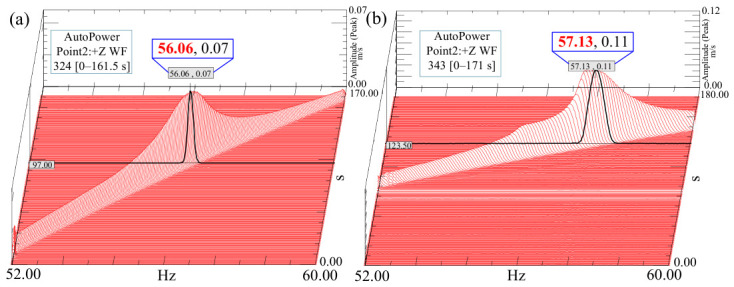
3D waterfall plots under sweep-frequency excitation within the range of the second natural frequency of CMCTP before and after coating under 800 °C (**a**) with coating, (**b**) without coating.

The measured natural frequencies of such specimens are determined by extracting the peak response frequencies from the sweep-frequency waterfall plots, as illustrated in Figure 4. Comparative analysis of predicted versus experimental natural frequencies for both coated and uncoated specimens under room- and elevated-temperature conditions is detailed in Table 2. With a maximum frequency calculation error of merely 4.7%, the numerical model exhibits strong agreement with experimental data, validating the predictive capability for vibrational characteristics. It should be noted that three specimens are used for the comparison at 25 °C, 400 °C, and 800 °C, respectively. Each specimen is first tested in the uncoated state and then tested again after applying the proposed coating. This test design is adopted due to the high cost of CMCTP specimens and their good short-term high-temperature resistance within the investigated range. The same fixture, torque-controlled clamping, and temperature fluctuation within ±3 °C are used to improve test consistency. A comparison between the calculated and measured mode shapes of the uncoated CMCTP at room temperature is presented in Table 3, demonstrating excellent agreement. As demonstrated in our prior findings [32], the small coating thickness and the temperature exhibit negligible influence on modal deformation patterns. Accordingly, the coated CMCTP exhibits mode shapes consistent with those of the uncoated reference specimen, with this characteristic preserved throughout both room-temperature and elevated-temperature working conditions.

**Figure 5:**
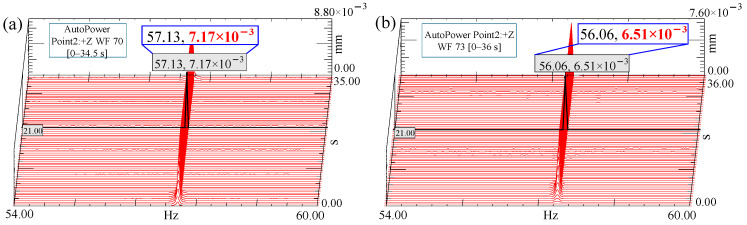
3D waterfall plots of the 2nd resonant response of CMCTP before and after coating under 800 °C (**a**) without coating, (**b**) with coating.

In pursuit of rigorous model validation, resonant responses at the measurement point obtained by fixed-frequency excitation at corresponding natural frequencies in Figure 5 are compared with theoretical predictions, with comparative results summarized in Table 4. Results demonstrate a maximum relative error of 8.3% in resonant amplitude prediction, demonstrating the reliability of the model for comprehensive vibration analysis. To reduce the influence of measurement variability, the frequency response and resonant response measurements are repeated three times under each condition. The mean values are used for comparison, and the maximum deviations are less than 0.5% for natural frequencies and less than 2% for resonant response amplitudes. These discrepancies listed in Table 2 and Table 4 may be attributed to uncertainties in temperature-dependent material properties, idealized uniform temperature distribution, and temperature fluctuation. Regarding model sensitivity, the predicted natural frequencies are mainly affected by the elastic moduli, coating thickness, and coefficients of thermal expansion, because these parameters determine the effective stiffness and thermal stress state of the coated CMCTP. The resonant responses are more sensitive to damping-related parameters.

### 3.4. Evaluation of Thermal Vibration Suppression of Predictive Coating

**Figure 6:**
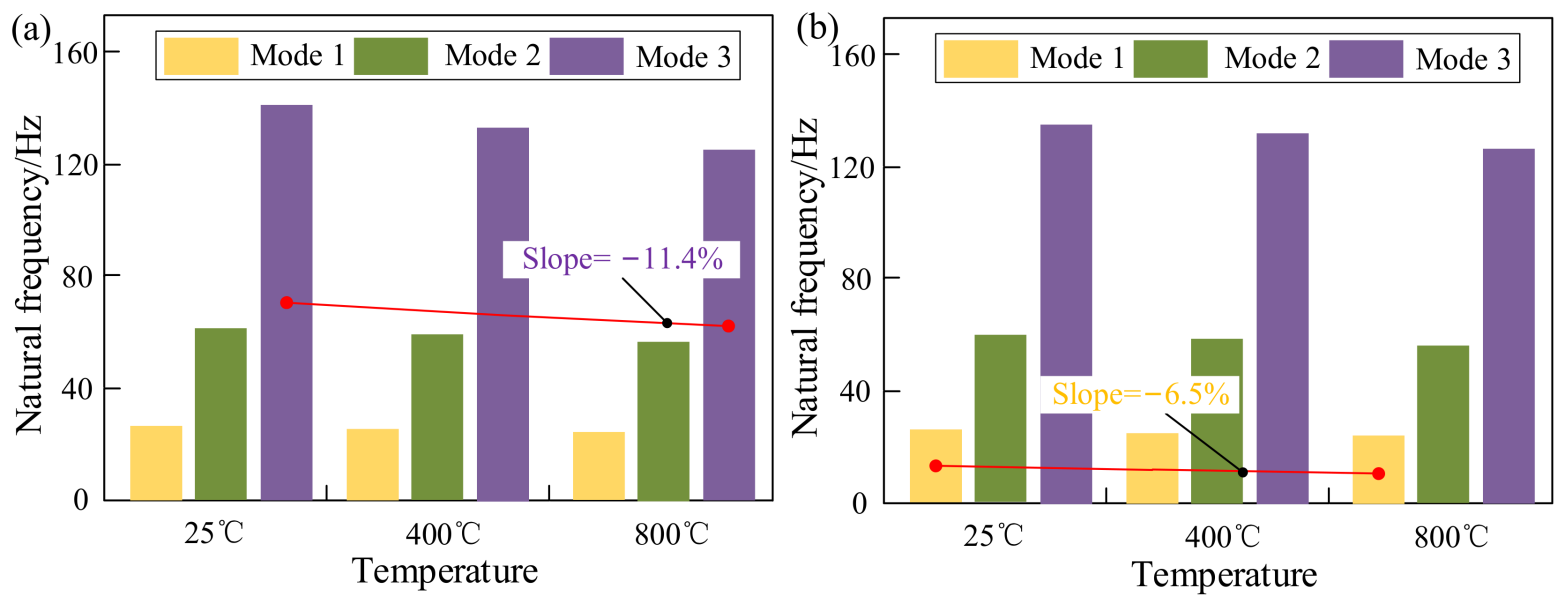
The first three natural frequencies of CMCTP under different temperatures (**a**) without coating, (**b**) with coating.

The vibration characteristics of the CMCTP are investigated from 25 °C to 800 °C to evaluate the effects of temperature on its dynamic characteristics. The variations in the natural frequencies in CMCTP as a function of temperature change are presented in Figure 6. As shown, the natural frequencies of the first three modes exhibit a monotonic decrease with increasing temperature, where the uncoated CMCTP exhibits a maximum reduction of 11.4% on the third natural frequency at 800 °C compared to room temperature, while the coated CMCTP shows a maximum decrease of 6.5% on the first natural frequency under identical thermal conditions. The decrease in natural frequency with increasing temperature is mainly associated with the thermally induced stress state in the coated CMCTP. Under the constrained boundary condition, temperature rise and thermal expansion mismatch between the coating and substrate generate internal thermal stresses, which modify the effective stiffness of the plate and lead to a reduction in natural frequencies.

**Figure 7:**
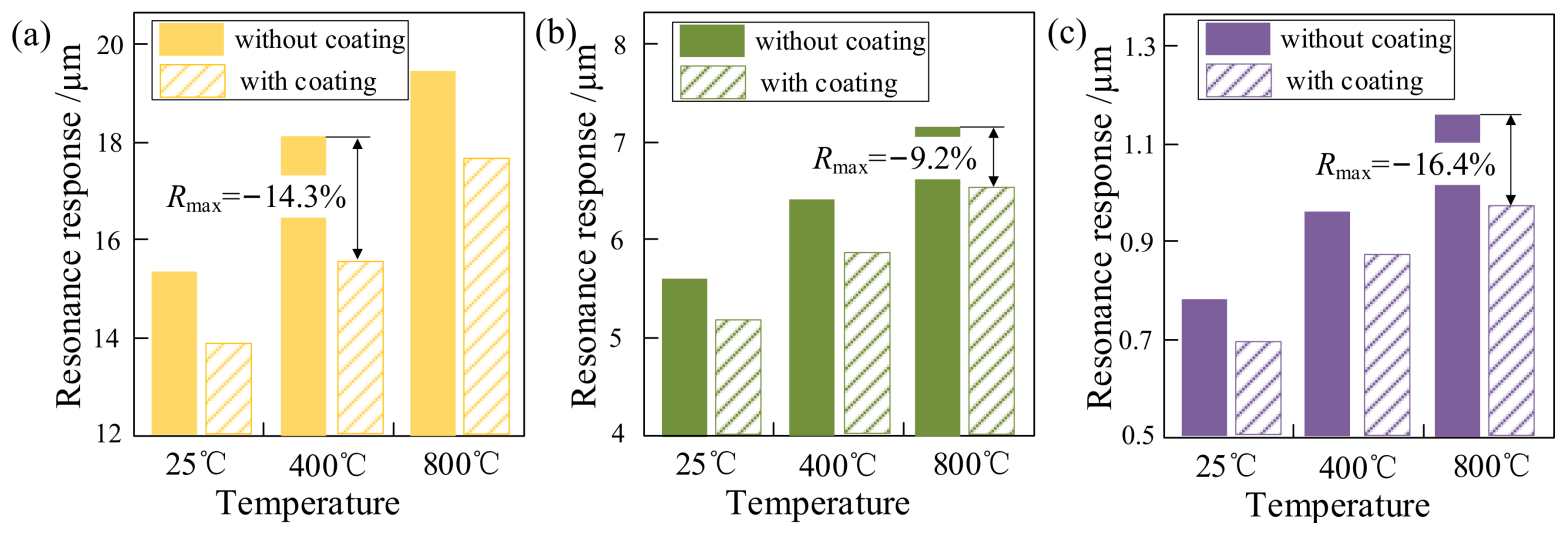
The resonance responses of coated and uncoated CMCTP under different temperatures: (**a**) Mode 1, (**b**) Mode 2, (**c**) Mode 3.

The effect of the protective coating on the resonant responses of CMCTP is systematically evaluated under 25 °C to 800 °C. As demonstrated in Figure 7, the coating significantly reduces resonant response amplitudes across all modes at elevated temperatures, demonstrating a maximum reduction of 14.3% for the first mode at 400 °C and 9.2% and 16.4% for the second and third modes at 800 °C, respectively. The observed reduction in resonant response is mainly attributed to the enhanced damping provided by the protective coating. Under the perfect bonding assumption, the coating deforms together with the CMCTP substrate and dissipates part of the vibration energy owing to its higher material loss factor, thereby increasing the effective damping of the coated CMCTP.

### 3.5. Preliminary Comparison with a Commercial Coating

**Figure 8:**
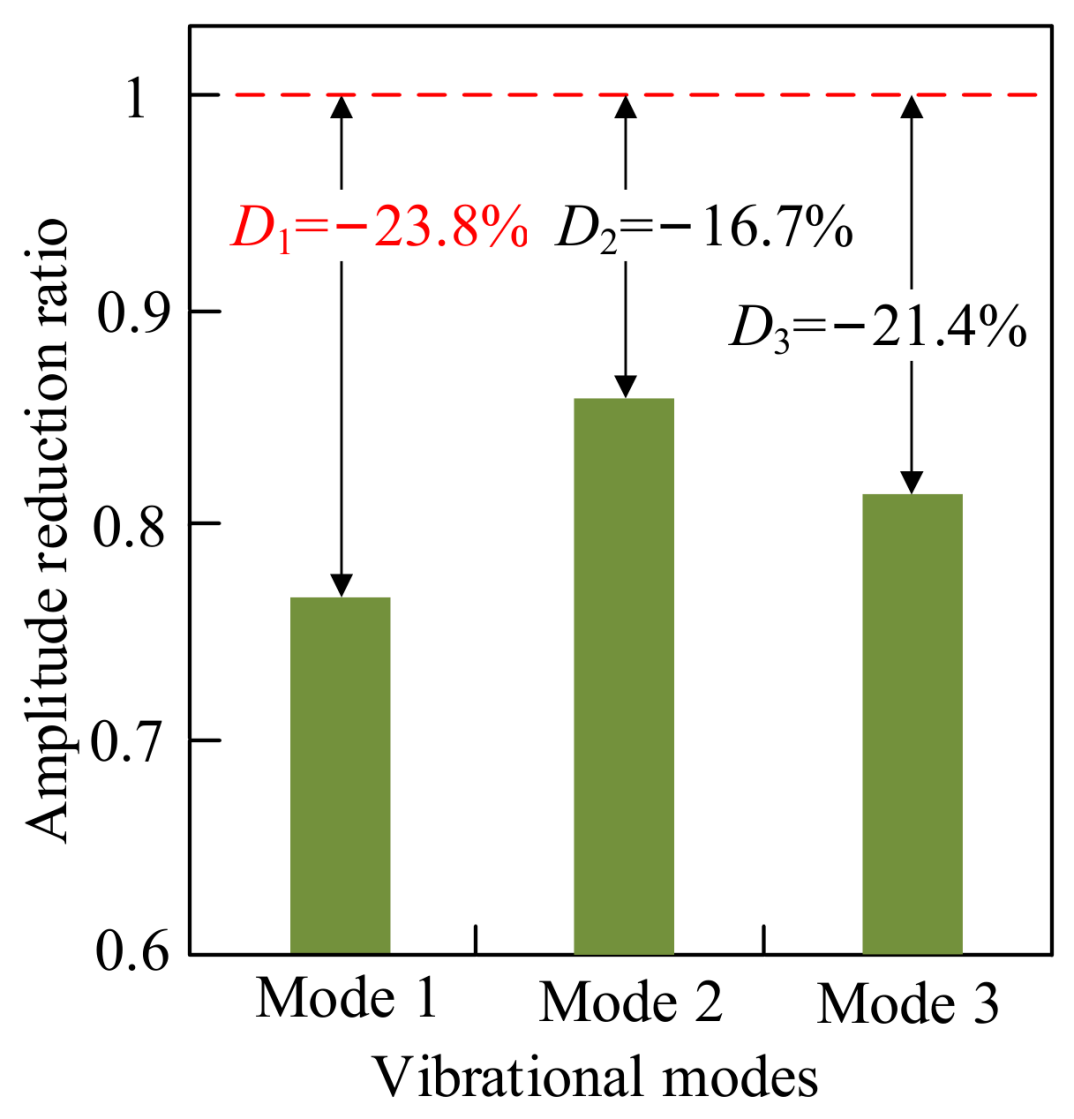
Relative vibration suppression efficacy of the proposed protective coating compared to the commercial coating across the first three resonant modes at 800 °C.

To further evaluate the engineering potential of the proposed protective coating, a preliminary comparison is conducted with ZS-1021, a commercially available high-temperature protective coating supplied by Beijing ZhiSheng WeiHua Chemical Co., Ltd. (Beijing, China). The proposed coating and ZS-1021 are applied at the same nominal thickness of 0.3 mm. An additional specimen coated with ZS-1021 is tested at 800 °C under the same cantilever boundary condition, excitation amplitude of 2.0 g, and response measurement position as those used for the proposed coating. The resonant response amplitudes of the first three modes are compared, as shown in Figure 8. The red dashed line at a normalized amplitude of 1 represents the response of the ZS-1021-coated specimen. Under the present test conditions, the proposed coating shows lower resonant response amplitudes than ZS-1021, with reductions of approximately 23.8%, 16.7%, and 21.4% for the first, second, and third modes, respectively. It should be noted that this comparison is based on one ZS-1021 coated specimen and is not repeated with multiple specimens. Therefore, the results are presented only as a preliminary comparison rather than a statistically conclusive assessment. Further tests with additional specimens are required to evaluate the reproducibility of this comparison.

## 4. Numerical Results and Discussion

The experimentally validated model provides the basis for parametric investigation of the vibration characteristics of the CMCTP. Unless otherwise specified, all calculated parameters maintain their default values, as defined in Section 3.1. To facilitate comparison among different parameter cases, both natural frequencies and resonant response amplitudes are normalized with respect to their corresponding reference values in the following analysis.

### 4.1. Effect of Coating-to-Substrate Thickness Ratio on Thermal Vibration Characteristics

**Figure 9:**
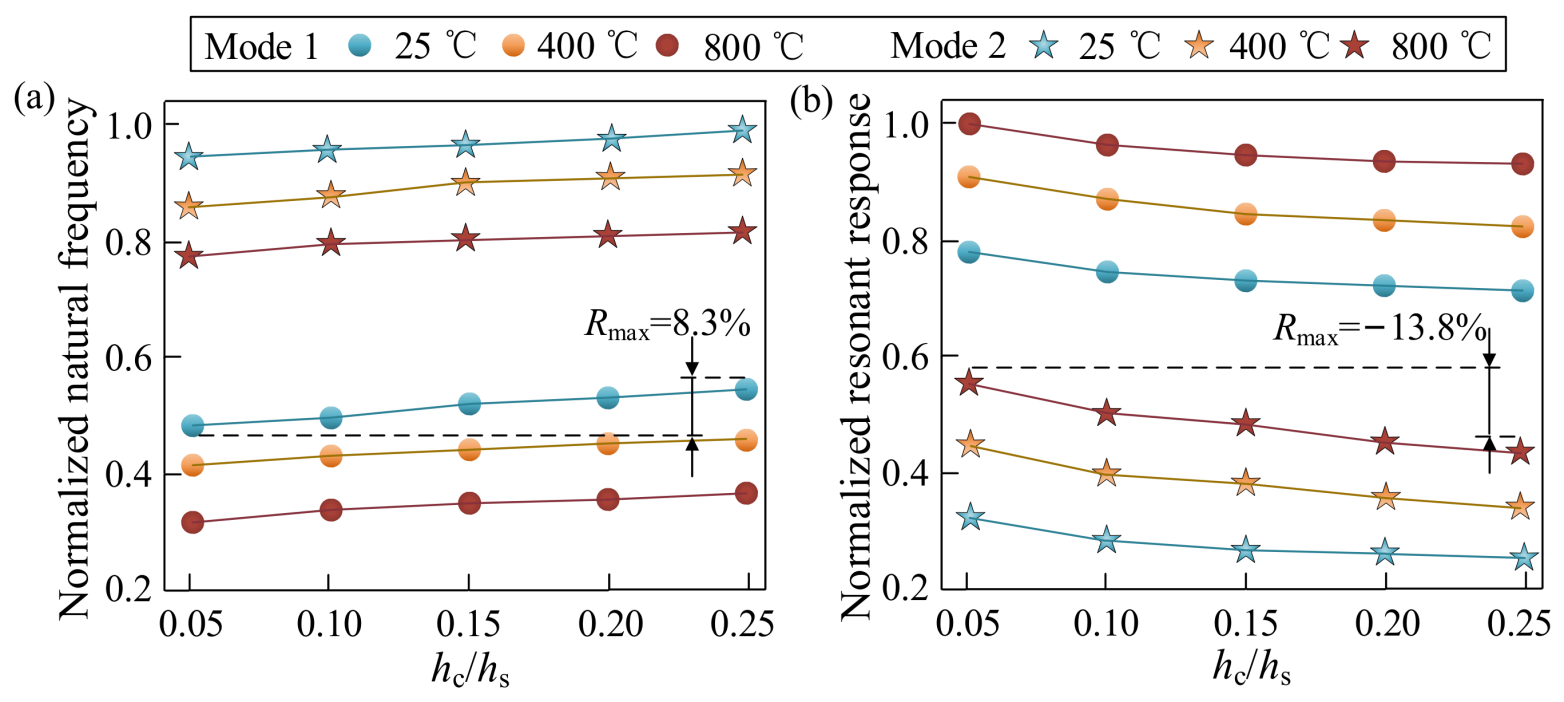
Effect of *h*_c_/*h*_s_ on the dynamic behaviors of a CMCTP under different temperature conditions: (**a**) normalized natural frequency, (**b**) normalized resonant response.

The influence of the coating-to-substrate thickness ratio *h*_c_/*h*_s_ from 0.05 to 0.25 on the dynamic performance of CMCTP is systematically investigated under thermal loading. As evidenced in Figure 9, both the normalized modal frequencies and harmonic vibration amplitudes of the CMCTP with protective coating exhibit distinct thickness-dependent trends at different temperatures, with frequencies increasing gradually while amplitudes decrease sharply as *h*_c_/*h*_s_ rises. For instance, the normalized first-mode natural frequency at 25 °C increases by up to 8.3% with increasing *h*_c_/*h*_s_, while a substantial reduction of 13.8% is observed in the normalized second-mode resonant response amplitude at 800 °C. The increase in natural frequencies is primarily attributed to the protective coating-induced bending stiffness enhancement, with comparatively negligible mass addition effects. In contrast, the resonant amplitude attenuation is mainly attributed to the increase in structural damping induced by the protective coating. For optimal thermo-vibrational suppression, a relatively large *h*_c_/*h*_s_ is recommended, considering coating process capabilities and structural weight limitations. Increasing *h*_c_/*h*_s_ enhances the participation of the coating in structural deformation, which increases the effective bending stiffness and damping contribution of the coated plate.

### 4.2. Effect of Coating-to-Substrate Modulus Ratio on Thermal Vibration Characteristics

**Figure 10:**
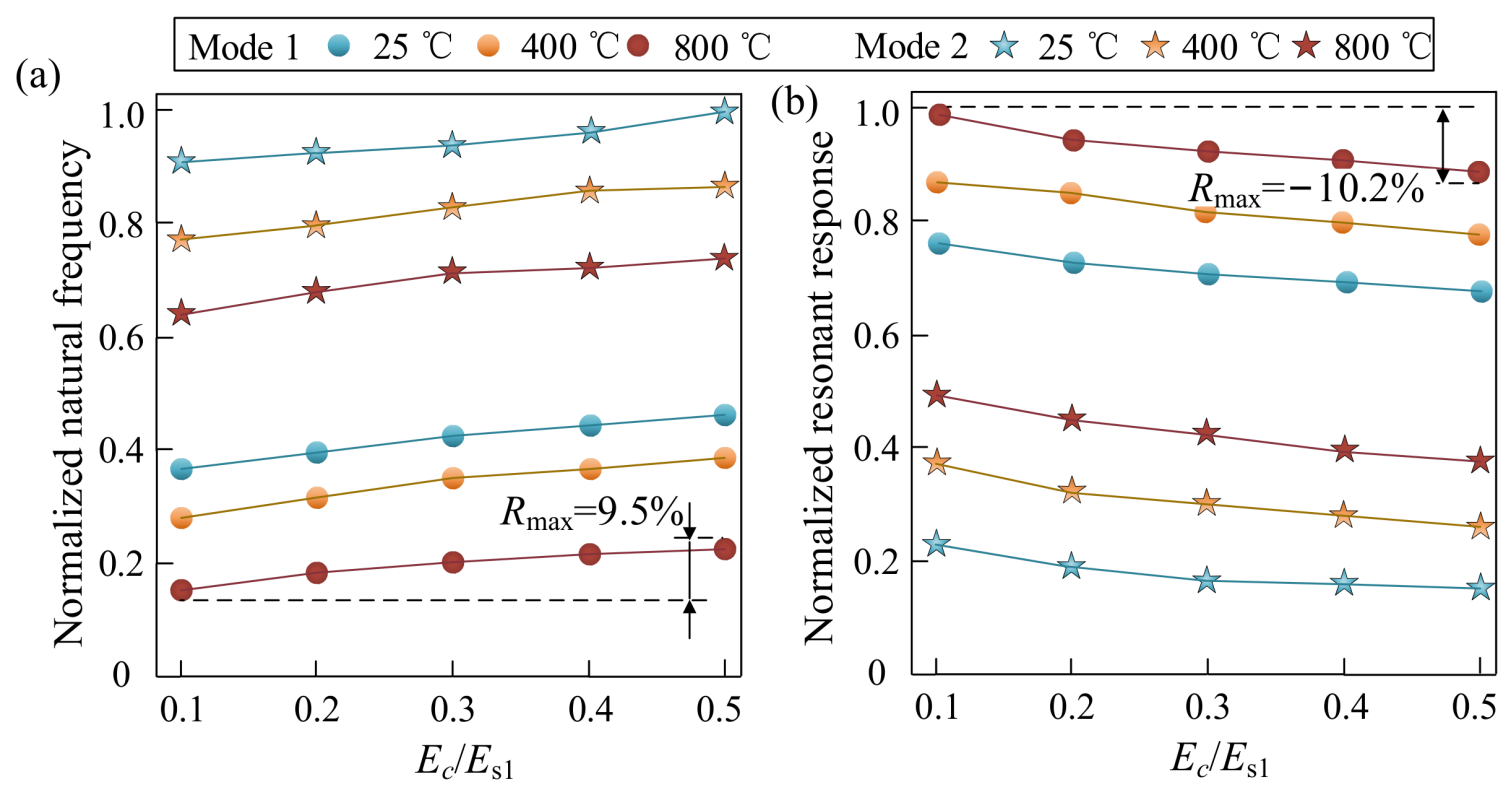
Effect of *E*_c_/*E*_s1_ on the dynamic behaviors of a CMCTP under different temperature conditions: (**a**) normalized natural frequency, (**b**) normalized resonant response.

The influence of the coating-to-substrate elastic modulus ratio *E*_c_/*E*_s1_ from 0.1 to 0.5 on the dynamic characteristics of CMCTP is investigated in various thermal conditions. As demonstrated in Figure 10, the normalized natural frequencies exhibit a monotonic increase with increasing *E*_c_/*E*_s1_, demonstrating maximum sensitivity at the first mode at 800 °C, with an increment of 9.5%. Conversely, the normalized resonant response amplitudes decrease significantly for all three modes, reaching a peak attenuation of 10.2% at the first mode at 800 °C. The higher *E*_c_/*E*_s1_ increases the structural natural frequencies by enhancing bending stiffness while simultaneously reducing its resonant responses through improving the structural effective damping induced by the protective coating. Comparative analysis reveals the superior damping efficacy of such a protective coating to suppress vibration under different temperatures. Therefore, a relatively large *E*_c_/*E*_s1_ within technological constraints (e.g., interfacial adhesion limits) is advised to optimize the thermo-vibrational suppression of such a structure. A larger *E*_c_/*E*_s1_ increases the stiffness contribution of the coating, thereby improving the resistance to resonant vibration.

### 4.3. Effect of Coating-to-Substrate Thermal Expansion Coefficient Ratio on Thermal Vibration Characteristics

**Figure 11:**
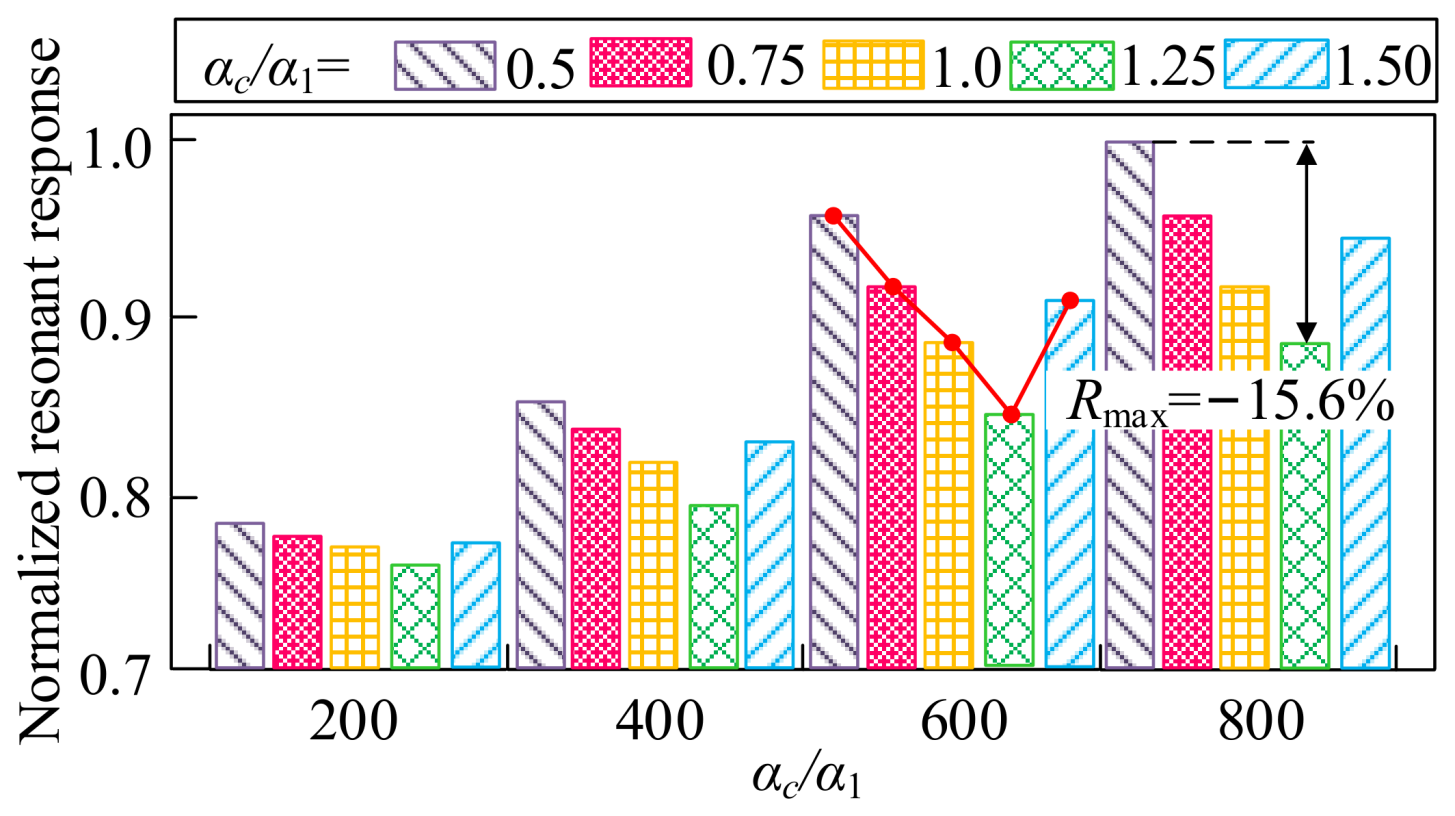
Effect of *α*_c_/*α*_1_ on the dynamic behaviors of a CMCTP under different temperature conditions. The red solid line connects the corresponding data points to illustrate the variation trend.

The influence of the coating-to-substrate thermal expansion coefficient ratio *α*_c_/*α*_1_ from 0.5 to 1.5 on the dynamic characteristics of CMCTP is investigated in various thermal conditions. The normalized resonant response amplitude of the first mode for the coated CMCTP under varying temperatures and *α*_c_/*α*_1_ ratios is presented in Figure 11. With increasing temperature, the influence of the *α*_c_/*α*_1_ ratios on the structural resonant response becomes more pronounced. The resonant amplitude reaches its minimum at *α*_c_/*α*_1_ = 1.25, showing a reduction of 15.6% compared to the case of *α*_c_/*α*_1_ = 0.5. These results suggest that the thermal expansion coefficient of the protective coating should be slightly higher than that of the substrate to maintain a mild compressive stress state of such a coating in a thermal environment. This effect is mainly associated with the thermally induced stress state arising from the mismatch in thermal expansion between the coating and the substrate. Therefore, appropriate matching of *α*_c_/*α*_1_ is important for coating design, and functionally graded coatings may be considered when a single coating material cannot meet the desired thermal expansion requirement.

## 5. Conclusions

A prediction method is developed to analyze the vibration characteristics of a CMCTP with the protective coating under high-temperature conditions, and its validity is verified through systematic experiments. Then, a comprehensive parametric study is conducted to evaluate the effects of key coating parameters on the dynamic response of the CMCTP. The main conclusions are summarized as follows:

(1) The prediction method demonstrates reliable predictive capability for both coated and uncoated CMCTP under high-temperature conditions, with maximum relative prediction errors of 4.7% for natural frequencies and 8.3% for resonant response amplitudes across the first three vibration modes.

(2) The protective coating reduces the resonant response amplitude by up to 16.4% for the third mode at 800 °C, primarily because of the increased effective structural damping provided by the coating. Furthermore, in the preliminary comparison with the commercial coating, the proposed coating reduces the resonant response amplitudes by 23.8%, 16.7%, and 21.4% for the first, second, and third modes at 800 °C, respectively.

(3) Within the investigated parameter ranges, the thermo-vibrational resistance of the coated CMCTP improves with the coating-to-substrate thickness and modulus ratios. The coating-to-substrate thermal expansion coefficient ratio also has a significant effect on the response, with the minimum resonant response amplitude occurring at an optimal ratio of 1.25.

The proposed model provides guidance for coating optimization and may be further combined with material degradation and damage evolution models for life prediction and health monitoring applications of high-temperature composite structures. However, the present study focuses on the modal frequencies and harmonic vibration responses of coated CMCTPs under uniform high-temperature conditions, without accounting for temperature nonuniformity or material degradation at elevated temperatures. In future research, these influencing factors will be systematically incorporated into the analysis.

## Figures and Tables

**Table 1:** Geometric and material properties of the investigated CMCTP and the coating at room temperature.

Type	Key Parameters
CMCTP	*E*_1_ = 36.8 GPa, *E*_2_ = 2.6 GPa, *G*_12_ = G_13_ = 1.9 GPa, G_23_ = 1.3 GPa, *ρ* = 2046 kg/m^3^, *υ*_1_ = 0.28, *η*_1_ = 0.0052, *η*_2_ = 0.0079, *η*_12_ = 0.0086, *α*_1_ = 6.0 × 10^−6^/^°^C, *α*_2_ = 7.2 × 10^−6^/^°^C, 170 mm × 125 mm × 2 mm (unclamped specimens)
Coating	*E*_c_ = 6.2 GPa, $\mu_{c}=0.3$, *ρ*_c_ = 1250 kg/m^3^, *h*_c_ = 0.3 mm, *η*_c_ = 0.0095, *α*_c_ = 7.5 × 10^−6^/^°^C

**Table 2:** The comparison of theoretical and experimental natural frequencies.

Coating	Mode Num.	25 °C			400 °C			800 °C		
		Calculation (Hz)	Experiment (Hz)	Error (%)	Calculation (Hz)	Experiment (Hz)	Error (%)	Calculation (Hz)	Experiment (Hz)	Error (%)
No	1	27.2	26.8	1.5	25.9	25.5	1.6	25.0	24.4	2.5
	2	62.8	61.3	2.4	61.3	59.4	3.2	59.0	57.1	3.3
	3	144.3	141.4	2.1	137.7	133.8	3.0	130.5	125.3	4.2
Yes	1	26.7	26.1	2.3	26.0	25.2	3.2	25.3	24.5	3.3
	2	61.7	59.7	3.4	60.6	58.9	3.0	58.3	56.1	3.9
	3	140.3	135.6	3.4	137.6	132.5	3.9	132.8	126.8	4.7

**Table 3:** The calculated and tested modal shapes of the CMCTP without coating under room temperature.

Mode Num.	1	2	3	4
Calculated results	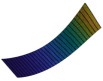	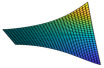	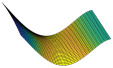	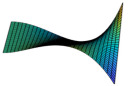
Tested results	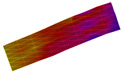	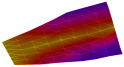	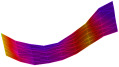	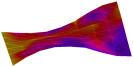

**Table 4:** Comparison of the theoretical resonant responses with the experimental ones.

Coating	Mode Num.	25 °C			400 °C			800 °C		
		Calculation (µm)	Experiment (µm)	Error (%)	Calculation (µm)	Experiment (µm)	Error (%)	Calculation (µm)	Experiment (µm)	Error (%)
No	1	16.05	15.34	4.6	18.98	18.13	4. 8	20.51	19.46	5.4
	2	5.96	5.62	6.0	6.82	6.42	6.2	7.52	7.17	4.9
	3	0.82	0.78	5.1	1.01	0.96	5.6	1.23	1.16	6.1
Yes	1	14.67	13.85	5.9	16.63	15.52	7.2	19.10	17.64	8.3
	2	5.49	5.17	6.2	6.23	5.85	6.5	7.03	6.51	7.7
	3	0.74	0.69	6.8	0.93	0.87	7.1	1.05	0.97	7.9

## Data Availability

The raw data supporting the conclusions of this article will be made available by the authors on request.

## Appendix A

The off-axis reduced stiffness coefficients of the CMCTP

(A1) $$\begin{array}{l} {\bar{Q}}_{11}^{s}=Q_{11}\cos^{4}\theta_{k}+2(Q_{12}+2Q_{66})\sin^{2}\theta_{k}\cos^{2}\theta_{k}+Q_{22}\sin^{4}\theta_{k}\\ {\bar{Q}}_{12}^{s}=(Q_{11}+Q_{22}-4Q_{66})\sin^{2}\theta_{k}\cos^{2}\theta_{k}+Q_{12}\left(\sin^{4}\theta_{k}+\cos^{4}\theta_{k}\right)\\ {\bar{Q}}_{22}^{s}=Q_{11}\sin^{4}\theta_{k}+2(Q_{12}+2Q_{66})\sin^{2}\theta_{k}\cos^{2}\theta_{k}+Q_{22}\cos^{4}\theta_{k}\\ {\bar{Q}}_{16}^{s}=(Q_{11}-Q_{12}-2Q_{66})\sin\theta_{k}\cos^{3}\theta_{k}+(Q_{12}-Q_{22}+2Q_{66})\sin^{3}\theta_{k}\cos\theta_{k}\\ {\bar{Q}}_{26}^{s}=(Q_{11}-Q_{12}-2Q_{66})\sin^{3}\theta_{k}\cos\theta_{k}+(Q_{12}-Q_{22}+2Q_{66})\sin\theta_{k}\cos^{3}\theta_{k}\\ {\bar{Q}}_{66}^{s}=(Q_{11}+Q_{22}-2Q_{12}-2Q_{66})\sin^{2}\theta_{k}\cos^{2}\theta_{k}+Q_{66}\left(\sin^{4}\theta_{k}+\cos^{4}\theta_{k}\right)\\ {\bar{Q}}_{44}^{s}=Q_{44},{\bar{Q}}_{55}^{s}=Q_{55} \end{array}$$

where *θ_k_* denotes an angle of the *k*-th layer fiber relative to the *x*-axis of the global coordinate system, and the elastic constants of the CMCTP are

(A2) $$\begin{array}{l} Q_{11}=\frac{E_{1}}{1-\nu_{12}\nu_{21}},Q_{22}=\frac{E_{2}}{1-\nu_{12}\nu_{21}},Q_{12}=\frac{\nu_{21}E_{1}}{1-\nu_{12}\nu_{21}},\\ Q_{44}=G_{23},Q_{55}=G_{13},Q_{66}=G_{12},\nu_{21}=\nu_{12}\frac{E_{2}}{E_{1}} \end{array}$$

where *E*_1_ and *E*_2_ are principal directions and Young’s moduli. *G*_12_ denotes in-plane shear modulus; *ν*_12_ and *ν*_21_ represent the Poisson’s ratios in the 1 and 2 directions.

The elastic constants of the protective coating are

(A3) $$Q_{11}^{c}=Q_{22}^{c}=\frac{E_{c}}{1-\nu_{c}^{2}},Q_{12}^{c}=\frac{\nu_{c}E_{c}}{1-\nu_{c}^{2}},Q_{44}^{c}=Q_{55}^{c}=Q_{66}^{c}=\frac{E_{c}}{2(1-\nu_{c})}$$

where *E*_c_ and $\nu_{c}$ denote the Young’s moduli and Poisson’s ratio of coating.
